# Integrative precision oncology in neuroblastoma: multi-omics biomarkers, molecular targets, and immunotherapeutic strategies

**DOI:** 10.3389/fonc.2026.1879586

**Published:** 2026-07-22

**Authors:** Amitabh Singh, Neha Goel, Sumit Mehndiratta, Aanchal Chaudhry, Manpreet Kaur, Sandeep Kumar Swain, Prashant Prabhakar, Aroonima Misra, Bhavika Rishi

**Affiliations:** 1 Department of Pediatrics, Division of Pediatric Oncology, Vardhman Mahavir Medical College and Safdarjung Hospital, New Delhi, India; 2 Department of Molecular Pathology, Indian Council of Medical Research (ICMR) - National Institute of Child Health Research, New Delhi, India

**Keywords:** liquid biopsy, multi-omics, MYCN, neuroblastoma, pediatric oncology, precision oncology, prognostic biomarkers, tumor microenvironment

## Abstract

Neuroblastoma is the most frequent extracranial solid tumor of young children and demonstrates a wide range of biological behavior, from spontaneous regression to highly aggressive metastatic disease. Recent advances in genomics, epigenetics, tumor microenvironment biology, and multi-omics technologies have greatly enhanced understanding of neuroblastoma pathogenesis and enabled the development of precision diagnostic and prognostic approaches. This review examines current evidence for molecular and cellular mechanisms underlying neuroblastoma initiation, progression, and therapeutic resistance, with a focus on MYCN amplification, ALK pathway dysregulation, chromosomal instability, telomere maintenance mechanisms, epigenetic remodeling, and neural crest developmental biology. This review primarily emphasizes that these characteristics should be interpreted within a unified developmental framework. In this context, age serves more as an indicator of the differentiation status of the sympathoadrenal progenitor pool rather than merely a timeline for the accumulation of somatic mutations. This viewpoint connects the epigenetic, genetic, and clinical aspects of the disease, providing insight into its distinctive range of behaviors, from spontaneous regression in infants to fatal progression in older children. The promising role of integrative diagnostic platforms — such as genomic profiling, liquid biopsy, radiomics, metabolomics, single-cell sequencing, and artificial intelligence-aided imaging approaches — in early detection and disease characterization is explored in detail. The evaluation of both existing and emerging prognostic models involves a critical analysis of molecular biomarkers, tumor microenvironment signatures, circulating tumor DNA, immune profiling, and machine-learning-based stratification systems. Additionally, the translational potential of systems biology approaches is examined, along with future directions for integrating multi-omics data into clinically relevant precision oncology frameworks. Integrative approaches are collectively reshaping neuroblastoma research by connecting molecular pathogenesis with advanced diagnostic and prognostic tools. These developments are expected to facilitate enhanced risk-adapted therapeutic strategies, personalized medicine, and ultimately, improved survival rates in children with neuroblastoma.

## 1 Introduction

Neuroblastoma is the most common extracranial solid tumor of childhood and one of the most biologically heterogeneous pediatric malignancies. It develops from primitive sympathoadrenal progenitor cells derived from the neural crest and encompasses a wide spectrum of clinical presentations, from spontaneous regression and differentiation to benign ganglioneuroma to rapidly progressive metastatic disease with poor survival (1, 2). Despite significant progress in multimodal therapeutic approaches, high-risk neuroblastoma remains responsible for a disproportionate share of global pediatric cancer mortality, with almost half of all cases classified as high-risk (1, 3). Neuroblastoma represents approximately 8–10% of all pediatric malignancies and accounts for nearly 15% of pediatric cancer-related fatalities, underscoring its considerable clinical and public health significance (1, 3). The disease affects mainly infants and young children, with a median age at diagnosis of approximately 17 months; more than 90% of cases are diagnosed before age 5, and it carries a slight male preponderance (2, 4). Epidemiological analyses reveal significant geographic and socioeconomic variations in incidence, mortality, and disability-adjusted life years (DALYs), highlighting inequalities in diagnostic infrastructure, access to specialist oncology care, and therapeutic outcomes across regions (1).

The clinical behavior of neuroblastoma is highly heterogeneous and is modulated by complex interactions between developmental biology, genomic instability, epigenetic dysregulation, and tumor microenvironment remodeling (5). Historically, prognostication has been driven largely by clinicopathological parameters such as age at diagnosis, disease stage, histopathological classification, and MYCN amplification status (6). Increasing knowledge of molecular heterogeneity, however, has fundamentally reshaped the understanding of neuroblastoma biology. Diverse oncogenic drivers and prognostic biomarkers have been identified — including ALK mutations, ATRX alterations, TERT rearrangements, segmental chromosomal aberrations, epigenetic signatures, and immune microenvironment-associated pathways — which collectively influence tumor progression, therapeutic resistance, and survival outcomes (5, 7, 8).

Contemporary therapeutic regimens incorporating intensive chemotherapy, surgery, radiotherapy, autologous stem cell transplantation, differentiation therapy, and anti-GD2 immunotherapy have improved survival in selected patient subsets; however, outcomes for children with high-risk and relapsed disease remain unsatisfactory (9). Treatment resistance, relapse, treatment-related toxicities, and intratumoral heterogeneity remain important clinical challenges (5, 9). Furthermore, conventional diagnostic techniques relying primarily on histopathology and imaging are increasingly recognized as insufficient to fully characterize the dynamic molecular complexity and evolutionary behavior of neuroblastoma.

The advancement of high-throughput sequencing technologies, single-cell transcriptomics, spatial biology, liquid biopsy platforms, radiomics, and artificial intelligence-based predictive modeling has initiated a transition from traditional pathology-based classification to integrative systems biology approaches in neuroblastoma research (7, 10, 11). These technologies allow multidimensional characterization of tumor genomics, epigenomics, metabolomics, immune landscapes, and clonal evolution, leading to more precise diagnostic stratification and personalized prognostic assessment. Integrative multi-omics approaches are being increasingly investigated to identify clinically actionable biomarkers, predict treatment response, monitor minimal residual disease, and optimize risk-adapted treatment strategies (10, 11).

This review presents a focused discussion of the current understanding of neuroblastoma pathogenesis, with particular emphasis on the molecular, epigenetic, and tumor microenvironmental mechanisms that drive disease heterogeneity. Novel integrative diagnostic and prognostic approaches — including genomic profiling, liquid biopsy, radiomics, and artificial intelligence-assisted strategies — are also discussed, along with their translational implications for advancing precision pediatric oncology. Instead of listing each new platform, we focus on a central theme: how the developmental origin of neuroblastoma influences its genetic and epigenetic characteristics, and how these characteristics, in turn, affect its clinical manifestations. We conduct a critical assessment of recent developments in multi-omics, radiomics, and artificial intelligence, distinguishing between those that have been integrated into clinical practice and those that are still in the exploratory phase.

## 2 Embryological origin and molecular pathogenesis

### 2.1 Neural crest development and tumor initiation

Neuroblastoma is a developmental malignancy derived from primitive neural crest-derived sympathoadrenal progenitor cells of the autonomic nervous system (ANS) (5). Neural crest cells (NCCs) tightly control their migration, proliferation, commitment to a specific lineage, and terminal differentiation during embryogenesis to give rise to sympathetic ganglia and adrenal medullary tissues. Dysregulation of these developmental pathways leads to the persistence of immature progenitor populations with the potential for malignant transformation (12, 13). Molecular and transcriptomic data increasingly support the notion that neuroblastoma is a disorder of abnormal embryonic development characterized by impaired sympathoadrenal maturation and lineage plasticity (10, 14).

The sympathoadrenal developmental program is controlled by highly coordinated transcriptional networks involving PHOX2B, HAND2, GATA3, ASCL1, and SOX11 (15). PHOX2B functions as a master regulator of sympathetic neuronal identity and ANS differentiation (16). Germline mutations in PHOX2B are strongly associated with familial neuroblastoma predisposition syndromes and congenital central hypoventilation syndrome, underscoring the pivotal role of this gene in neural crest biology (16). HAND2 and GATA3 cooperate to maintain adrenergic lineage identity and control catecholaminergic differentiation pathways (17). More recent studies have demonstrated that ASCL1 is implicated in neuroendocrine differentiation and cellular plasticity, whereas SOX11 expression is enriched in undifferentiated high-risk neuroblastoma phenotypes and is associated with aggressive tumor behavior (18, 19).

Single-cell transcriptomic studies have considerably advanced the understanding of neuroblastoma ontogeny. The resemblance of high-risk neuroblastoma cells to fetal sympathoblast populations supports the “developmental arrest hypothesis” — the notion that tumor cells are arrested in immature embryonic states as a consequence of defective differentiation signaling (10, 20). This developmental arrest creates intratumoral heterogeneity, promotes metastatic dissemination, and contributes to therapy resistance. Defective neural crest migration and persistence of progenitor-like cellular programs are increasingly identified as drivers of tumor initiation (13).

Schwannian stromal interactions are also important in neuroblastoma biology. Stromal-rich tumors with abundant Schwannian stroma are typically associated with favorable histology and better clinical prognosis, whereas stromal-poor tumors show aggressive proliferative phenotypes (21). Schwann cells secrete neurotrophic factors, extracellular matrix proteins, and mediators that promote differentiation and inhibit tumor progression (22). These findings highlight the importance of developmental microenvironmental interactions in determining neuroblastoma behavior and prognosis.

### 2.2 Genomic alterations in neuroblastoma

#### 2.2.1 MYCN amplification

MYCN amplification is one of the most extensively studied genomic drivers in neuroblastoma, occurring in approximately 20–25% of cases and predominantly in high-risk disease (5, 23). It is strongly correlated with rapid tumor progression, metastatic dissemination, treatment resistance, and poor survival outcomes independent of disease stage (6). MYCN is a transcriptional regulator that drives oncogenesis through broad reprogramming of cellular proliferation, metabolism, ribosomal biogenesis, and differentiation pathways (24). MYCN-amplified tumors show accelerated cell cycle progression through activation of cyclin-dependent kinases and inhibition of apoptotic signaling pathways (25). Furthermore, MYCN promotes ribosomal RNA synthesis and protein translation to sustain increased biosynthetic and proliferative demands (26).

More recent evidence has also clarified the role of MYCN in metabolic reprogramming. Increased glycolytic flux, glutamine dependence, nucleotide biosynthesis, and mitochondrial adaptation in MYCN-driven neuroblastoma cells allow tumor survival under metabolically stressed conditions (27). These metabolic vulnerabilities have emerged as therapeutic targets in aggressive neuroblastoma.

#### 2.2.2 ALK mutations

Anaplastic lymphoma kinase (ALK) alterations are the most common actionable mutations described in neuroblastoma (28). Germline activating ALK mutations are involved in hereditary neuroblastoma predisposition syndromes, and somatic mutations are frequently found in sporadic and relapsed disease (29). Common hotspot mutations — including F1174L, R1275Q, and F1245C — lead to constitutive kinase activation and downstream oncogenic signaling (28). ALK signaling activates several oncogenic pathways, including PI3K/AKT/mTOR, RAS/MAPK, and JAK/STAT cascades, thereby promoting tumor proliferation, survival, angiogenesis, and metastatic progression (30). Functional cooperation between ALK activation and MYCN amplification further drives aggressive tumor phenotypes and poor clinical outcomes (31).

Recognition of ALK as a therapeutic target has significantly advanced precision medicine approaches for neuroblastoma. Clinical trials of ALK inhibitors, including crizotinib, lorlatinib, and ceritinib, are underway for relapsed and refractory disease (32). However, secondary resistance mutations and activation of bypass pathways remain important therapeutic challenges.

#### 2.2.3 Segmental chromosomal alterations

Neuroblastoma is characterized by recurrent segmental chromosomal aberrations of major diagnostic and prognostic relevance (29). Loss of chromosome 1p is strongly associated with MYCN amplification and aggressive tumor biology, likely reflecting loss of tumor suppressor genes involved in apoptosis and genomic stability (30). Deletion of chromosome 11q is another important prognostic abnormality, frequently found in high-risk non-MYCN-amplified neuroblastoma and characterized by pronounced chromosomal instability, defective DNA damage response pathways, and worse survival (30). Gain of chromosome 17q is the most frequent chromosomal abnormality in neuroblastoma and is strongly associated with advanced-stage disease and poor prognosis (29).

ATRX alterations are most commonly detected in older children and adolescents with neuroblastoma and are associated with defects in chromatin remodeling, genomic instability, and treatment resistance (31). Most ATRX-mutated tumors exhibit alternative lengthening of telomeres (ALT), which may underlie the chronic relapsing nature of the disease in this subgroup.

#### 2.2.4 Telomere maintenance mechanisms

Telomere maintenance has emerged as a defining hallmark of aggressive neuroblastoma biology (31). In high-risk tumors, telomere stabilization is achieved either through TERT rearrangements that activate telomerase or through ALT mechanisms associated with ATRX dysfunction (31). TERT rearrangements cause aberrant telomerase expression and cellular immortalization, whereas ALT-positive tumors maintain telomere length via homologous recombination-mediated pathways (31). Activation of telomere maintenance mechanisms is strongly correlated with metastatic disease, therapeutic resistance, and poor overall survival. Emerging data suggest that telomere biology may serve as both a prognostic biomarker and a potential therapeutic vulnerability in neuroblastoma.

**Table 1** summarizes the major genomic alterations in neuroblastoma and their diagnostic and prognostic significance.

**Table 1:** Major genomic alterations in neuroblastoma and their diagnostic/prognostic significance.

Genomic alteration	Biological mechanism	Clinical significance	Therapeutic relevance	References
MYCN amplification	Increased proliferation, metabolic reprogramming, stemness maintenance	Strongly associated with high-risk disease and poor survival	Indirect targeting via BET inhibitors; synthetic lethality strategies	(5, 19, 23, 24)
ALK mutation/amplification	Constitutive receptor tyrosine kinase activation	Aggressive disease biology; familial predisposition	Targetable with ALK inhibitors (lorlatinib, crizotinib)	(24, 28, 29)
1p deletion	Loss of tumor suppressor genes	Associated with unfavorable prognosis	Used in molecular risk stratification	(8, 30)
11q deletion	Genomic instability and defective DNA repair	Poor prognosis in non-MYCN-amplified tumors	Potential DNA damage response-targeted therapies	(30)
17q gain	Enhanced oncogenic signaling	Associated with advanced-stage disease	Prognostic biomarker	(29)
ATRX mutation	Chromatin remodeling defects and ALT activation	Common in older children; relapsing disease course	Potential vulnerability to DNA repair-targeted therapies	(8, 31)
TERT rearrangement	Telomerase activation and cellular immortalization	Strong association with aggressive disease	Telomere-directed therapeutic strategies under investigation	(8, 31)

ALK, anaplastic lymphoma kinase; ALT, alternative lengthening of telomeres; ATRX, alpha-thalassemia/mental retardation syndrome X-linked; MYCN, MYCN proto-oncogene; TERT, telomerase reverse transcriptase. The listed major genomic alterations are among the most clinically relevant molecular abnormalities currently incorporated into neuroblastoma biological risk assessment and precision therapeutic investigation (5, 8, 19, 24–31).

### 2.3 Epigenetic dysregulation

Epigenetic dysregulation, in conjunction with genomic alterations, plays a crucial role in the initiation, progression, and therapeutic resistance of neuroblastoma (31). Abnormal DNA methylation leads to the silencing of tumor suppressor genes and the preservation of undifferentiated cellular states (31). Hypermethylation of genes involved in differentiation and apoptotic regulation has been associated with aggressive clinical behavior and poor outcomes.

Histone modification pathways and chromatin remodeling complexes are key determinants of neuroblastoma transcriptional plasticity (15). Aberrant activity of histone methyltransferases such as EZH2 promotes oncogenic transcription through trimethylation of histone H3K27 and repression of differentiation-associated genes (15). Furthermore, EZH2 overexpression has been associated with metastatic progression, stemness-associated phenotypes, and immune evasion.

Super-enhancer biology has also been identified as a key determinant of neuroblastoma lineage identity (12, 15). MYCN, HAND2, GATA3, and PHOX2B form a core transcriptional regulatory circuitry that cooperates through super-enhancer-associated chromatin landscapes to maintain adrenergic tumor states (12, 15). Therapeutic exploitation of these transcriptional dependencies with bromodomain and epigenetic inhibitors is being explored as a promising anti-tumor approach (26).

Non-coding RNAs represent another important layer of epigenetic regulation in neuroblastoma (12). The miR-17–92 cluster is transcriptionally activated by MYCN and promotes proliferation while inhibiting apoptosis (24). Conversely, miR-34a functions as a tumor suppressor and is frequently downregulated in aggressive disease. Suppression of let-7 microRNAs by LIN28B also maintains stem cell-like properties and tumor aggressiveness (12).

Long non-coding RNAs (lncRNAs) such as NBAT1 and CASC15 have been shown to regulate cellular differentiation, invasion, and treatment response. These epigenetic mechanisms collectively contribute to neuroblastoma heterogeneity and therapeutic resistance.

### 2.4 Tumor microenvironment and immune evasion

The neuroblastoma tumor microenvironment (TME) is increasingly recognized as a key determinant of tumor progression, metastatic dissemination, and therapeutic response (31). High-risk neuroblastomas are generally characterized by highly immunosuppressive TMEs with prominent tumor-associated macrophages (TAMs), cancer-associated fibroblasts (CAFs), regulatory T cells, and inhibitory cytokine networks (22, 31).

TAMs promote tumor progression by secreting IL-6, TGF-β, VEGF, and extracellular matrix-remodeling enzymes that enhance angiogenesis, immune escape, and metastatic dissemination (31). Studies have consistently shown that increased macrophage infiltration correlates with poor survival and resistance to immunotherapy (44). CAFs also contribute to stromal fibrogenesis, extracellular matrix remodeling, and establishment of pro-tumorigenic signaling niches (22).

Dysfunction of natural killer (NK) cells is another important mechanism of immune escape in neuroblastoma (31). Tumor cells downregulate activating ligands and secrete immunosuppressive cytokines that blunt NK cell-mediated cytotoxicity. Chronic antigenic stimulation also leads to T-cell exhaustion with upregulation of inhibitory receptors such as PD-1, TIM-3, and LAG-3 (31).

Neuroblastoma cells release extracellular vesicles, including exosomes, that further modulate immunosuppression by transferring oncogenic proteins, microRNAs, and immunoregulatory molecules. These vesicles contribute to metastatic niche formation, angiogenesis, and therapeutic resistance.

The translational relevance of TME biology has become increasingly evident in the era of immunotherapy (31). Anti-GD2 monoclonal antibody therapy has substantially improved outcomes in high-risk neuroblastoma, but immunosuppressive TMEs and heterogeneous immune activation states limit therapeutic efficacy (31). New combinations of anti-GD2 antibodies with checkpoint inhibitors, cytokine modulation, NK cell therapies, and macrophage-targeted strategies are currently under active investigation.

Effective development of precision oncology strategies in neuroblastoma, therefore, requires an integrated understanding of the interplay between developmental biology, tumor genomics, epigenetic plasticity, and immune microenvironmental signaling.

### 2.5 An integrated developmental framework: age, epigenetic state, and clonal architecture

The preceding sections describe MYCN amplification, ALK signaling, telomere maintenance, segmental chromosomal change, and epigenetic dysregulation as if they were parallel tracks; in neuroblastoma, they are better understood as facets of a single developmental problem. In most adult carcinomas, older age is largely a surrogate for the time available to accumulate somatic mutations. Neuroblastoma does not follow this logic. Although mutational burden does rise modestly with age, pan-neuroblastoma sequencing shows that clock-like and reactive-oxygen-associated mutational signatures, together with TERT- and ATRX-related telomere-maintenance lesions, are enriched in older children, whereas MYCN amplification and near-triploid, structurally simpler genomes dominate in infants (48) — the total number of coding mutations remains among the lowest of any human cancer (8). The clinically decisive variable is therefore not how long mutations have accumulated, but the developmental state of the sympathoadrenal progenitor at which transformation occurs.

This developmental state is held open by the epigenetic machinery described above. The adrenergic core regulatory circuitry (MYCN, PHOX2B, HAND2, GATA3) and Polycomb repressive complex 2 (PRC2), acting through EZH2-mediated trimethylation of histone H3K27, together maintain an immature, proliferative identity and silence the differentiation and tumor-suppressor programs that would otherwise allow a neuroblast to mature (13, 15); MYCN reinforces this repression by recruiting PRC2. In infancy, the sympathoadrenal lineage still contains abundant immature progenitors whose developmental programs are incompletely silenced and remain poised either to differentiate or to involute. This poised epigenetic state is the most plausible explanation both for the spontaneous regression and maturation characteristic of stage MS (4S) and many infant tumors, and for the favorable prognosis of children younger than 18 months (5, 6). In older children, the developmental window has effectively closed: transformation arises in a more constrained epigenetic landscape and is accompanied by the stabilizing genetic lesions — activation of telomere maintenance, 11q loss, ATRX disruption — that distinguish aggressive, regression-resistant disease, consistent with the observation that tumors engaging a telomere-maintenance mechanism in the context of RAS or p53 pathway alterations follow the most lethal course (7). Age, epigenetic plasticity, and genotype are thus three perspectives on a single developmental axis rather than independent prognostic factors.

**Figure 1:**
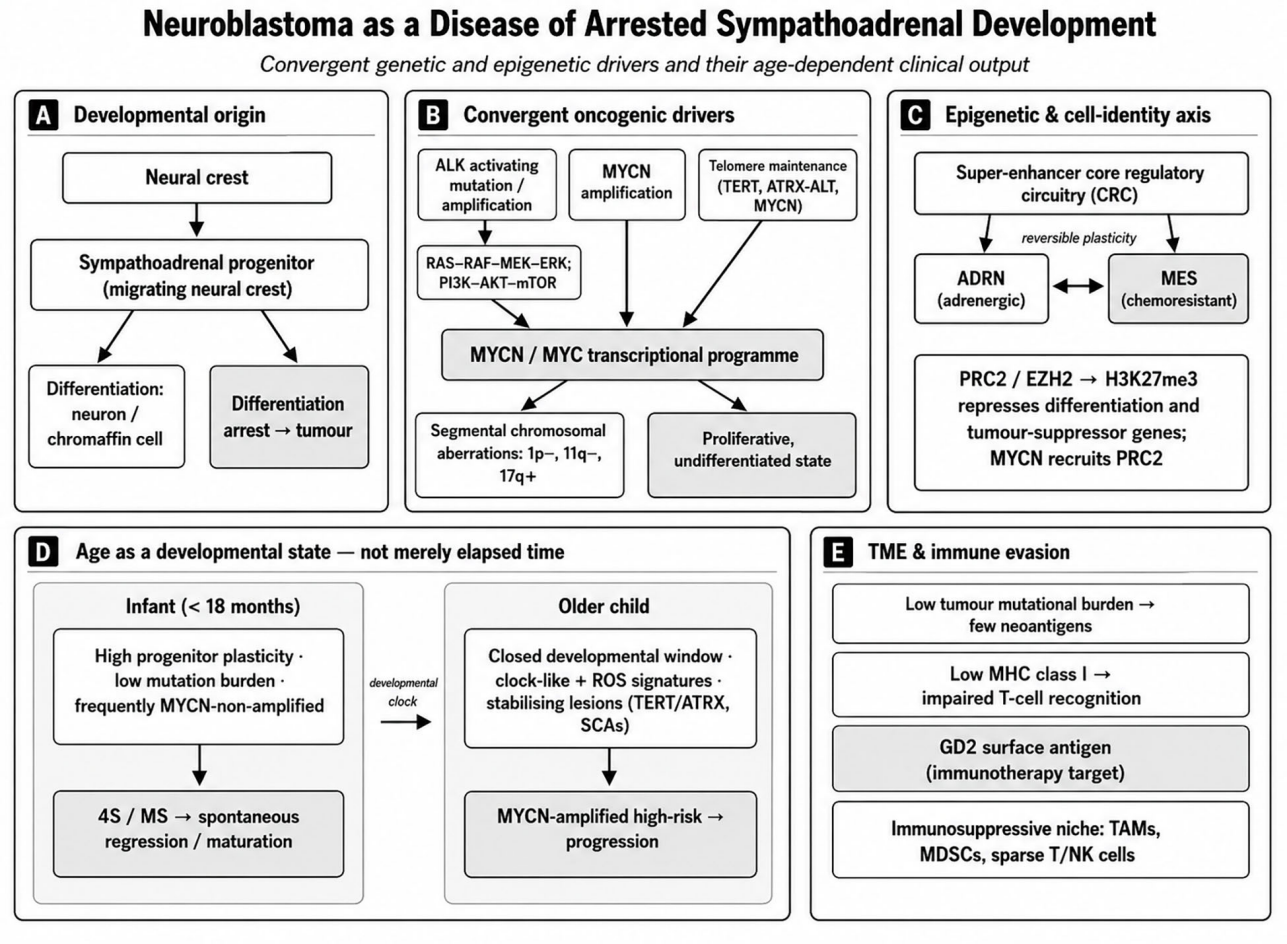
Integrated molecular pathways driving neuroblastoma pathogenesis. **(A)** Developmental origin. **(B)** Convergent oncogenic drivers. **(C)** Epigenetic and cell-identity axis. **(D)** Developmental framework of age-dependent disease behavior. **(E)** Tumor microenvironment and immune evasion. ADRN, adrenergic; ALK, anaplastic lymphoma kinase; ALT, alternative lengthening of telomeres; CRC, core regulatory circuitry; EZH2, enhancer of zeste homolog 2; GD2, disialoganglioside 2; MDSCs, myeloid-derived suppressor cells; MES, mesenchymal; MHC, major histocompatibility complex; MYC, MYC proto-oncogene; MYCN, MYCN proto-oncogene; PRC2, Polycomb repressive complex 2; TAMs, tumor-associated macrophages; TERT, telomerase reverse transcriptase; TME, tumor microenvironment.

Reading these drivers as a developmental program also clarifies how they should be interpreted clinically, because not every alteration is present in every tumor cell. In a Children’s Oncology Group study of high-risk tumors sequenced at diagnosis, canonical RAS–MAPK pathway drivers, including ALK mutations, were frequently subclonal rather than truncal, whereas MYCN amplification and segmental copy-number changes were typically clonal (49). A subclonal driver is unlikely to be eradicated by single-agent inhibition of its target and may instead permit outgrowth of resistant populations — a mechanism that helps explain the limited durability of upfront ALK-directed monotherapy and that reinforces the case, developed in later sections, for clonality-aware biomarker interpretation and serial monitoring rather than single-time-point, single-region testing (49). Throughout the remainder of this review, we return to this framework, asking of each genomic, epigenetic, liquid-biopsy, and imaging biomarker not only whether it is associated with outcome but whether it reflects a truncal developmental event that can realistically guide therapy. These interconnected molecular, epigenetic, and microenvironmental pathways are summarized in **Figure 1**.

## 3 Integrative diagnostic approaches

### 3.1 Conventional diagnostic modalities

The correct diagnosis and risk stratification of neuroblastoma are based on the integration of histopathological, biochemical, radiological, and molecular findings (5, 6). Although precision oncology has revolutionized modern diagnostics of pediatric cancer, assessment and therapeutic planning in neuroblastoma still rely substantially on traditional modalities.

Histopathological examination remains the gold standard for diagnosis confirmation (17). Neuroblastoma exhibits a wide histological spectrum from undifferentiated neuroblastic tumors to mature ganglioneuromatosis with abundant Schwannian stroma (5). Histology is classified according to the International Neuroblastoma Pathology Classification (INPC) or Shimada classification, which considers the degree of neuroblastic differentiation, Schwannian stromal development, mitosis-karyorrhexis index (MKI), and patient age to distinguish tumors into favorable and unfavorable histology (17). Favorable histology correlates with differentiated phenotypes and better survival, while unfavorable histology is strongly associated with aggressive biology, metastatic dissemination, and treatment resistance (5, 17).

Bone marrow evaluation plays an important role given the high incidence of metastatic marrow infiltration in high-risk neuroblastoma (5). Bilateral bone marrow aspiration (BMA) and trephine biopsy are routinely performed for staging and assessment of minimal residual disease (MRD). Morphologic evaluation with immunohistochemistry (IHC) and molecular assays provides increased sensitivity in detecting occult metastatic disease (31).

Biochemical evaluation through urinary catecholamine metabolites remains a hallmark diagnostic tool in neuroblastoma (5). Approximately 90–95% of tumors secrete catecholamines or their metabolites — in particular vanillylmandelic acid (VMA) and homovanillic acid (HVA) (4). Elevated urinary metabolites are useful not only for diagnosis but also as markers of tumor burden and response to therapy.

Imaging is instrumental in defining the extent of the primary tumor, metastatic dissemination, surgical resectability, and image-defined risk factors (IDRFs) (6). Conventional imaging modalities, including ultrasonography, computed tomography (CT), and magnetic resonance imaging (MRI), remain integral to initial evaluation (5). MRI offers superior soft tissue characterization without ionizing radiation exposure, which is particularly important in the pediatric population.

Functional imaging has transformed neuroblastoma staging. Metaiodobenzylguanidine (MIBG) scintigraphy is considered the standard modality of functional imaging given its high specificity for catecholaminergic tissues (31, 32). MIBG imaging is used for staging, assessment of metastatic spread, treatment monitoring, and relapse detection. However, approximately 10% of tumors exhibit low or absent MIBG avidity, particularly in poorly differentiated or recurrent lesions (32). In these cases, fluorodeoxyglucose positron emission tomography/computed tomography (FDG-PET/CT) provides an important complementary imaging modality (33).

### 3.2 Molecular diagnostics

The introduction of molecular diagnostics has transformed neuroblastoma classification from a largely morphology-based scheme to a biologically driven precision oncology framework (7). The convergence of genomic, transcriptomic, cytogenetic, and single-cell technologies has facilitated improved understanding of tumor heterogeneity, clonal evolution, and actionable therapeutic vulnerabilities.

#### 3.2.1 Genomic profiling

High-throughput genomic profiling has greatly advanced understanding of neuroblastoma biology (8). Whole-genome sequencing (WGS) enables comprehensive detection of structural rearrangements, chromosomal instability, copy-number alterations, and mutational landscapes (8). WGS studies have identified recurrent genomic aberrations associated with aggressive disease phenotypes, including MYCN amplifications, ALK mutations, ATRX alterations, and TERT rearrangements (8, 24, 25).

Whole exome sequencing (WES) interrogates primarily coding genomic regions and has enabled the identification of germline predisposition syndromes and therapeutically targetable somatic mutations (3, 34). The discovery of germline and somatic ALK mutations through exome sequencing has significantly advanced the development of targeted therapies for neuroblastoma (24, 25).

Targeted next-generation sequencing (NGS) panels are increasingly integrated into clinical workflows, allowing rapid and cost-effective detection of clinically actionable mutations (2). Panels currently in use include MYCN, ALK, ATRX, TP53, TERT, and other recurrently altered neuroblastoma genes (31). These approaches are particularly useful in relapsed and refractory disease, where therapeutic resistance is often driven by genomic evolution (35).

RNA sequencing has become a powerful transcriptomic tool for characterizing developmental programs, immune signatures, and cellular states (10). Transcriptomic profiling has identified distinct adrenergic and mesenchymal neuroblastoma phenotypes with differing therapeutic responses and survival outcomes (15, 36). These studies have revealed dramatic lineage plasticity and adaptive transcriptional reprogramming under therapeutic pressure.

#### 3.2.2 Cytogenetic techniques

Cytogenetic evaluation remains an important component of neuroblastoma diagnostics, as multiple chromosomal abnormalities carry substantial prognostic significance (29). Fluorescence *in situ* hybridization (FISH) for MYCN amplification is routinely performed in diagnostic laboratories, as MYCN amplification remains one of the strongest adverse prognostic biomarkers (19).

Array comparative genomic hybridization (array CGH) enables genome-wide detection of copy number alterations and segmental chromosomal abnormalities (29). This technology has greatly improved detection of recurrent alterations, including 1p deletion, 11q deletion, and 17q gain, all of which are linked to aggressive tumor behavior (30).

Single-nucleotide polymorphism (SNP) arrays further improve the characterization of chromosomal instability, copy-neutral loss of heterozygosity, and subclonal genomic alterations (29). These technologies have advanced the understanding of neuroblastoma clonal evolution and genomic complexity.

#### 3.2.3 Single-cell technologies

Single-cell technologies have revolutionized the understanding of neuroblastoma heterogeneity and developmental biology (10). Single-cell RNA sequencing (scRNA-seq) allows high-resolution characterization of tumor cellular architecture, developmental trajectories, and tumor microenvironment interactions (10, 14).

Recent scRNA-seq studies have demonstrated the resemblance of neuroblastoma cells to fetal sympathoblast populations, lending support to the developmental arrest hypothesis (10). Importantly, single-cell studies have revealed the coexistence of adrenergic and mesenchymal tumor cell states that can dynamically interconvert under therapeutic pressure (15, 36). Mesenchymal populations exhibit increased migratory capacity, stemness-associated properties, and chemotherapy resistance.

Single-cell technologies also enable the detection of rare resistant subclones that can drive relapse and disease progression (37). Mapping cellular heterogeneity at single-cell resolution, therefore, has major translational implications for personalized therapeutic stratification and real-time monitoring of tumor evolution. These emerging molecular diagnostic technologies, together with their underlying principles, clinical utility, and current limitations, are summarized in **Table 2**.

**Table 2:** Emerging molecular diagnostic technologies in neuroblastoma.

Technology	Principle	Clinical utility	Limitations	References
Whole genome sequencing (WGS)	Genome-wide mutation and structural variant analysis	Comprehensive genomic profiling	High cost; complex interpretation	(8)
Whole exome sequencing (WES)	Sequencing of coding regions	Detection of driver mutations and predisposition genes	Misses non-coding alterations	(3, 34)
Targeted NGS panels	Focused sequencing of actionable genes	Rapid molecular diagnostics	Limited genomic breadth	(2, 31)
RNA sequencing	Transcriptomic profiling	Identification of cellular states and immune signatures	Requires high-quality RNA	(10, 15, 36)
FISH for MYCN	Fluorescent probe-based amplification detection	Standard MYCN assessment	Limited multiplexing capability	(19)
Array CGH	Copy number variation analysis	Detection of chromosomal aberrations	Lower resolution than sequencing	(29, 30)
SNP arrays	Genome-wide polymorphism assessment	Detection of LOH and genomic instability	Limited functional interpretation	(29)
Single-cell RNA sequencing	Individual cell transcriptomic profiling	Cellular heterogeneity and resistant clone identification	Expensive and computationally intensive	(10, 14)
Liquid biopsy assays	Analysis of circulating tumor-derived material	Real-time disease monitoring	Sensitivity limitations in low tumor burden	(11, 38, 40)

Array CGH, array comparative genomic hybridization; ctDNA, circulating tumor DNA; FISH, fluorescence *in situ* hybridization; LOH, loss of heterozygosity; NGS, next-generation sequencing; RNA-seq, RNA sequencing; scRNA-seq, single-cell RNA sequencing; SNP, single-nucleotide polymorphism; WES, whole-exome sequencing; WGS, whole-genome sequencing. Technologies summarized include established and emerging molecular platforms used for genomic characterization, assessment of tumor heterogeneity, and precision oncology-based neuroblastoma diagnostics (8, 10, 11, 29, 37–42).

### 3.3 Liquid biopsy

Liquid biopsy technologies represent promising minimally invasive techniques for dynamic tumor monitoring and precision diagnostics in neuroblastoma (11). Standard tissue biopsy is limited in longitudinal evaluation of tumor evolution, response to therapy, and minimal residual disease, whereas liquid biopsy allows assessment of circulating tumor-derived material with greater convenience and repeatability.

Circulating tumor DNA (ctDNA) is among the most extensively investigated components of liquid biopsy (11). MYCN amplification, ALK mutations, segmental chromosomal alterations, and emerging resistance-associated mutations can be detected by ctDNA analysis (38, 39). Serial ctDNA monitoring may enable early detection of relapse before clinically overt radiological progression (38).

Circulating tumor cells (CTCs) have also been explored as biomarkers for metastatic dissemination and treatment response (40). The prognostic relevance of detecting neuroblastoma-associated transcripts in peripheral blood and bone marrow samples has been demonstrated in high-risk disease (37).

Neuroblastoma-derived exosomes contain oncogenic proteins, microRNAs, and immunoregulatory molecules that regulate tumor progression and immune escape (40). Exosomal microRNA signatures may serve as sensitive biomarkers for disease monitoring and relapse prediction. Cell-free RNA (cfRNA) analysis and methylation-based assays provide additional avenues for minimally invasive transcriptomic and epigenetic profiling (40), potentially increasing sensitivity in detecting minimal residual disease.

Collectively, liquid biopsy approaches offer significant advantages, including minimally invasive procedures, dynamic longitudinal monitoring, and a more comprehensive representation of tumor heterogeneity compared with single-site tissue biopsy (11, 40). There is growing interest in leveraging these technologies to track real-time clonal evolution, therapeutic resistance, and early relapse.

### 3.4 Advanced imaging and radiomics

Advanced imaging technologies have significantly improved neuroblastoma characterization, staging, assessment of treatment response, and prognostic evaluation (5). Conventional functional imaging modalities are increasingly complemented by radiomics and artificial intelligence (AI)-based analytical approaches capable of extracting quantitative imaging biomarkers.

MIBG scintigraphy remains the standard functional imaging modality because of its high specificity for catecholaminergic tumors (31, 32). MIBG imaging is critical for metastatic assessment, response evaluation, and relapse surveillance (5). However, MIBG-negative tumors — particularly in aggressive dedifferentiated disease — require alternative imaging strategies (32).

FDG-PET/CT has emerged as an important complementary imaging modality, especially in MIBG-negative or relapsed neuroblastoma (33). PET imaging provides valuable metabolic information and may improve the detection of residual or recurrent disease. MRI-based functional imaging techniques, including diffusion-weighted imaging (DWI) and dynamic contrast-enhanced MRI, are increasingly used for assessing tumor cellularity, vascularity, and therapeutic response (33). Apparent diffusion coefficient (ADC) values derived from DWI have been shown to correlate with tumor aggressiveness and treatment response.

Radiomics refers to the extraction of large-scale quantitative imaging features from radiographic datasets, including texture analysis, voxel-based heterogeneity mapping, and shape descriptors that provide insights into tumor biology beyond conventional visual interpretation (41). Emerging radiomic models have demonstrated a promising ability to predict MYCN amplification status, therapeutic response, and survival outcomes (42).

Artificial intelligence and deep learning-based imaging platforms are increasingly being integrated into pediatric oncologic imaging workflows (41). Automated machine learning algorithms can analyze complex imaging datasets to generate predictive risk models and support clinical decision-making. Integration of imaging features with genomic and transcriptomic data has given rise to the emerging field of radiogenomics (42), which aims to establish associations between imaging phenotypes and underlying molecular characteristics, facilitating non-invasive molecular risk stratification and personalized treatment planning.

Integrative diagnostic approaches combining molecular profiling, liquid biopsy technologies, advanced imaging, and AI-driven analytics are collectively redefining neuroblastoma management and accelerating the transition toward precision pediatric oncology.

## 4 Prognostic stratification and biomarker discovery

### 4.1 Current risk stratification systems

Neuroblastoma is marked by profound biological and clinical heterogeneity, ranging from spontaneous regression to highly aggressive metastatic disease resistant to multimodal therapy (5). Accurate prognostic stratification is therefore essential to optimize risk-adapted therapeutic approaches while minimizing treatment-related toxicity in low-risk patients (2, 6).

The International Neuroblastoma Risk Group (INRG) classification system is currently the most widely accepted framework for pretreatment risk stratification in pediatric oncology (6). The INRG system uses image-defined risk factors (IDRFs) to standardize pretreatment classification and therapy planning — a key distinction from prior staging systems that relied predominantly on post-operative findings (6). Based on the combination of clinical, pathological, radiological, and molecular parameters, patients are classified into very low-, low-, intermediate-, and high-risk groups (6).

Among prognostic variables, age at diagnosis remains one of the strongest independent predictors of outcome (5). Children younger than 18 months have better survival due to increased differentiation potential, higher prevalence of hyperdiploid tumors, and greater likelihood of spontaneous tumor regression (5, 6). In contrast, older children more frequently exhibit aggressive molecular phenotypes and therapy-resistant disease (31). As discussed in Section 2.5, this age effect is best interpreted developmentally rather than as a function of mutation accrual: infant tumors combine high epigenetic plasticity with structurally simpler, frequently MYCN-non-amplified genomes, whereas older children more often carry telomere-maintenance and segmental lesions and, importantly, their targetable RAS–MAPK drivers are frequently subclonal at diagnosis (48, 49).

Histopathological classification based on the INPC (Shimada system) further refines prognostication (17). The INPC combines degree of neuroblastic differentiation, Schwannian stromal development, MKI, and patient age to separate favorable from unfavorable histology (17). Tumors with favorable histology are associated with a rich Schwannian stroma that promotes differentiation and improves outcomes, whereas unfavorable histology is associated with aggressive proliferation, metastatic spread, and poor survival (17, 18).

MYCN amplification is the single most powerful negative molecular prognostic marker in neuroblastoma, occurring in approximately 20–25% of tumors (19). It is strongly associated with rapid tumor progression, metastatic dissemination, treatment resistance, and poor prognosis, independent of clinical stage (20, 21). DNA ploidy is also an important risk stratification factor; hyperdiploid tumors are generally associated with a favorable prognosis in younger children (6).

Segmental chromosomal abnormalities, such as 1p deletion, 11q deletion, and 17q gain, have major prognostic significance (29, 30). Tumors with 11q deletion, in particular, exhibit chromosomal instability and poor survival, independent of MYCN amplification status (30). Modern risk classification systems are increasingly incorporating these genomic alterations as supplements to traditional clinicopathological variables (2).

The Children’s Oncology Group (COG) risk stratification framework, widely used in North America, incorporates similar clinicobiological variables (2). COG protocols are increasingly incorporating molecular profiling, MRD assessment, and dynamic evaluation of therapeutic response to improve prognostic precision (2).

Despite substantial progress, current stratification systems remain constrained by intratumoral heterogeneity, temporal clonal evolution, and the inability to fully predict therapeutic resistance or relapse (35). These limitations have prompted the incorporation of multi-omics biomarkers, tumor microenvironment signatures, and AI-based predictive algorithms into next-generation prognostic models.

### 4.2 Molecular prognostic biomarkers

#### 4.2.1 Genomic biomarkers

Genomic biomarkers have revolutionized neuroblastoma prognostication and precision oncology approaches (8). The most established genomic biomarker remains MYCN amplification, which is strongly correlated with high-risk disease, rapid progression, and poor survival (19, 20). MYCN-driven tumors exhibit increased proliferative potential, metabolic reprogramming, and stemness-associated phenotypes, all contributing to aggressive clinical behavior (13, 14).

ATRX mutations are an important prognostic biomarker, especially in older children and adolescents (8). ATRX-deficient tumors frequently exhibit ALT, genomic instability, and chronic relapsing disease courses (31). These tumors are often characterized by slow proliferation kinetics but are associated with marked therapeutic resistance and poor long-term outcomes (35).

Structural rearrangements that activate TERT have become a well-recognized mechanism of telomere maintenance and tumor immortalization (8). TERT rearrangements are strongly associated with metastatic disease, aggressive biological phenotypes, and poor survival (31). Recent genomic studies indicate that telomere maintenance status may represent one of the most powerful integrated biomarkers of high-risk neuroblastoma (5).

ALK alterations are of both prognostic and therapeutic relevance (24, 25). ALK mutations activate PI3K/AKT/mTOR and RAS/MAPK signaling pathways, driving aggressive tumor behavior (26, 27). Synergistic functional cooperation between ALK activation and MYCN amplification further enhances oncogenic potential (27). Importantly, ALK-targeted therapies — including lorlatinib — have shown promising activity in relapsed and refractory neuroblastoma and are currently under evaluation in Phase 2/3 trials (28).

The mutational divergence between primary and relapsed tumors further highlights the complexity of neuroblastoma evolution (35). Relapsed tumors frequently acquire additional genomic alterations associated with therapeutic resistance and clonal selection, underscoring the importance of longitudinal molecular monitoring (35).

#### 4.2.2 Transcriptomic signatures

Transcriptomic profiling has greatly improved understanding of neuroblastoma heterogeneity and prognostic biology (7). Distinct adrenergic and mesenchymal transcriptional states have been identified, with mesenchymal phenotypes characterized by enhanced migratory capacity, stemness-related properties, immune evasion, and chemotherapy resistance (15, 36).

Super-enhancer-associated transcriptional circuitry involving MYCN, HAND2, GATA3, and PHOX2B is a major determinant of cellular identity in neuroblastoma (13, 15). Van Groningen et al. demonstrated that neuroblastoma comprises two major super-enhancer-associated differentiation states that can be dynamically interconverted under therapeutic pressure (43). Similarly, Gartlgruber et al. identified enhancer-driven regulatory subtypes correlating with distinct prognostic behavior and lineage plasticity (45).

Immune-related transcriptomic signatures have also emerged as important prognostic determinants (31). Tumors with increased cytotoxic T-cell infiltration, NK-cell activation, and interferon-related pathway activity are generally associated with better survival outcomes and higher immunotherapy response rates (31, 44).

Transcriptional programs associated with stemness are strongly linked to poor prognosis (13, 14). MYCN-driven tumors exhibit elevated expression of stem cell-related pathways, metabolic plasticity, and dedifferentiation-associated signatures that promote metastatic dissemination and therapeutic resistance (14).

#### 4.2.3 Epigenetic biomarkers

Epigenetic dysregulation is an important factor in neuroblastoma progression and prognostication (15). Aberrant DNA methylation patterns contribute to the silencing of tumor suppressor genes and the maintenance of undifferentiated cellular states (5). Hypermethylation signatures involving apoptosis-related and differentiation-associated genes have been associated with aggressive clinical behavior and poor outcomes (15).

Histone modification pathways also regulate tumor progression and lineage plasticity (13, 15). Dysregulated EZH2 activity drives stemness-associated transcriptional programs, metastatic dissemination, and immune evasion via histone H3K27 trimethylation (13). High EZH2 expression is a strong predictor of poor prognosis and therapy resistance.

MicroRNA (miRNA) signatures have been identified as promising minimally invasive prognostic biomarkers (15). MYCN-regulated miR-17–92 clusters promote proliferation and inhibit apoptosis, while aggressive neuroblastoma frequently downregulates tumor suppressor miRNAs such as miR-34a (20). Epigenetic and miRNA-based biomarkers are therefore important future tools for dynamic risk assessment and therapeutic monitoring.

### 4.3 Tumor microenvironment-based prognosis

The TME is increasingly recognized as a major determinant of neuroblastoma progression, metastatic spread, and treatment response (31). Immune and stromal signatures frequently carry prognostic significance independent of tumor genomic alterations.

Immune infiltration scores have become important prognostic biomarkers of survival and immunotherapeutic responsiveness (31). Tumors with higher proportions of activated cytotoxic T lymphocytes, NK cells, and antigen-presenting cells tend to have better outcomes (31). Conversely, immunosuppressive microenvironments characterized by TAMs, regulatory T cells, and inhibitory cytokine networks are associated with aggressive disease biology (44).

Macrophage polarization toward M2-like phenotypes contributes significantly to tumor progression by promoting angiogenesis, extracellular matrix remodeling, metastatic dissemination, and immune escape (44). Asgharzadeh et al. reported that infiltration of M2-polarized inflammatory cells in metastatic neuroblastoma is associated with poorer survival (44). Cytokine profiling has also been shown to be prognostic; elevated levels of IL-6, TGF-β, VEGF, and other pro-inflammatory mediators are associated with therapy resistance, stromal remodeling, and poor survival (31).

Stromal signatures also influence neuroblastoma behavior. Stromal-rich Schwannian tumors exhibit greater differentiation and favorable histology, whereas stromal-poor tumors are aggressive and exhibit proliferative phenotypes (17, 18). Cancer-associated fibroblasts also create signaling niches that promote tumor growth and modify the extracellular matrix (18).

Increasing recognition of TME-mediated therapeutic resistance has driven the development of integrated immune-genomic prognostic models to enhance individualized risk prediction and guide immunotherapy selection (31).

**Table 3** summarizes established and emerging prognostic biomarkers currently under investigation in neuroblastoma.

**Table 3:** Established and emerging prognostic biomarkers in neuroblastoma.

Biomarker category	Biomarker	Prognostic implication	Clinical application	Evidence level (references)
Genomic	MYCN amplification	Poor prognosis; aggressive disease	Risk stratification	Validated; routine (19, 20)
Genomic	ALK mutation	Therapy resistance and aggressive behavior	Targeted therapy selection	Validated; actionable (24, 28)
Genomic	ATRX mutation	Chronic relapsing disease; ALT activation	Relapse risk assessment	Established (8, 31)
Genomic	TERT rearrangement	High-risk metastatic phenotype	Prognostic stratification	Established (8, 31)
Cytogenetic	11q deletion	Genomic instability and poor survival	Risk classification	Validated; routine (30)
Cytogenetic	17q gain	Advanced disease stage	Prognostic assessment	Validated; routine (29)
Transcriptomic	Adrenergic/mesenchymal signatures	Therapy resistance and lineage plasticity	Molecular subtyping	Investigational (15, 36, 43)
Epigenetic	DNA methylation signatures	Tumor suppressor silencing	Prognostic biomarker development	Investigational (31)
Epigenetic	EZH2 overexpression	Stemness and immune evasion	Epigenetic therapeutic targeting	Preclinical (13)
miRNA	miR-17–92 cluster	Increased proliferation	Molecular prognostication	Investigational (20, 24)
Immune	Tumor-associated macrophages	Poor immunotherapy response	TME-based prognosis	Investigational (44)
Radiomic	PET/CT radiomics signatures	MYCN prediction and relapse risk	AI-assisted prognostic modeling	Investigational (42)
Liquid biopsy	ctDNA and exosomal biomarkers	Real-time tumor evolution monitoring	Minimal residual disease assessment	Emerging (11, 38, 40)

ALK, anaplastic lymphoma kinase; ALT, alternative lengthening of telomeres; ctDNA, circulating tumor DNA; EZH2, enhancer of zeste homolog 2; miRNA, microRNA; MYCN, MYCN proto-oncogene; PET/CT, positron emission tomography/computed tomography; TME, tumor microenvironment. The listed biomarkers are clinically validated and investigational prognostic markers associated with neuroblastoma risk stratification, tumor progression, therapeutic resistance, and survival outcomes (6–8, 15, 30, 31, 35, 43–45).

### 4.4 Artificial intelligence and predictive modelling

AI-based predictive modeling is increasingly transforming neuroblastoma prognostication and precision oncology (41). Machine learning approaches can integrate large-scale clinicopathological, genomic, transcriptomic, radiomic, and microenvironmental datasets to improve the accuracy of individualized prognostic prediction (41). These models can capture complex nonlinear relationships among biomarkers that are generally not identifiable by traditional statistical techniques. These capabilities are predominantly investigational, necessitating a cautious approach to the enthusiasm they generate. The majority of published neuroblastoma models are retrospective, originate from single institutions, and are limited in size. External validation is rare, and issues such as class imbalance and batch effects can artificially enhance perceived performance. Furthermore, few studies report model calibration or comply with established standards for prediction-model reporting. Until models are prospectively and externally validated, machine-learning outputs should be considered research tools rather than a foundation for individual treatment decisions.

Multi-omics integration represents one of the most important emerging directions in neuroblastoma prognostication (2). The combination of genomic, transcriptomic, epigenetic, radiomic, and liquid biopsy datasets enables comprehensive characterization of tumor biology and dynamic clonal evolution (10, 35).

Radiomics methods that extract quantitative imaging features from MRI, PET/CT, and MIBG have shown increasing prognostic utility (42). Qian et al. developed a PET/CT-based radiomics signature that predicts MYCN amplification status with promising diagnostic accuracy (42). Such imaging-genomic integration strategies may enable non-invasive molecular risk stratification.

Deep learning-based survival prediction models using imaging and histopathological datasets are also evolving rapidly (41). Integration of AI-assisted image analysis into digital pathology platforms is enabling increasingly reproducible quantification of stromal content, immune infiltration, MKI, and cellular heterogeneity (41). Predictive nomograms combining clinical, molecular, radiomic, and microenvironmental biomarkers are increasingly studied for individualized therapeutic planning (2).

**Figure 2:**
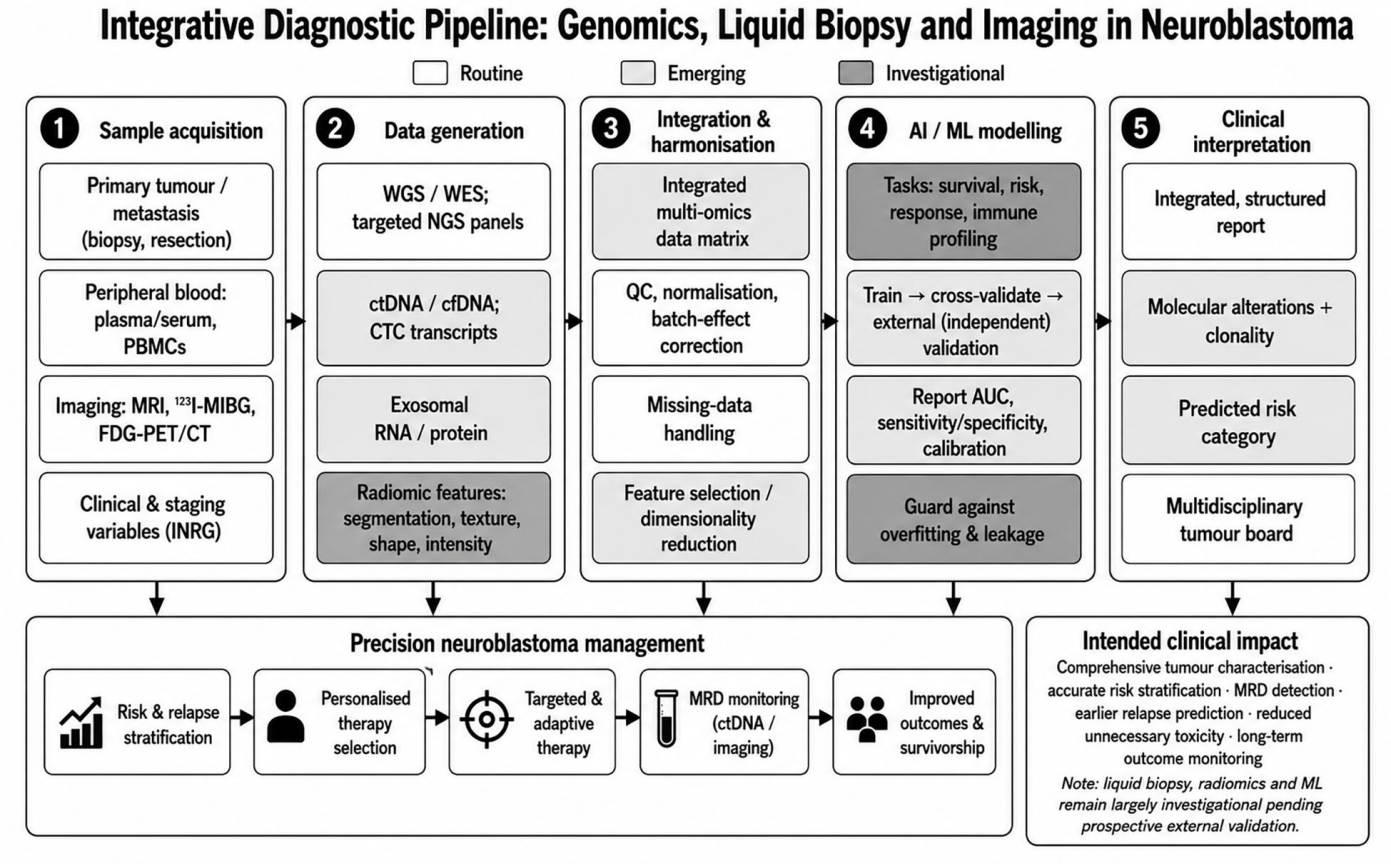
Integrative diagnostic pipeline combining genomics, liquid biopsy, and radiomics in neuroblastoma. The image depicts a multimodal workflow for neuroblastoma diagnostics, illustrating the complementary roles of tissue-based genomic profiling, liquid biopsy platforms (ctDNA, CTCs, exosomes), functional imaging (MIBG, FDG-PET/CT), and radiomics-AI integration in achieving comprehensive tumor characterization and precision risk stratification.

Advances in biomarker discovery, systems biology, and AI-driven analytics are converging to redefine neuroblastoma prognostication and accelerate the transition to precision pediatric oncology. This integrative diagnostic pipeline combining genomic profiling, liquid biopsy, functional imaging, and radiomics-AI integration is depicted in **Figure 2**.

## 5 Translational and therapeutic implications

### 5.1 Precision medicine approaches

Advances in knowledge of neuroblastoma developmental biology, genomic heterogeneity, epigenetic plasticity, and tumor microenvironmental interactions have substantially accelerated the shift toward precision oncology-based therapeutic strategies (2, 5). Current treatment paradigms are increasingly incorporating molecular risk stratification, dynamic biomarker assessments, and targeted therapeutic interventions to improve survival while minimizing treatment-related toxicity.

#### 5.1.1 Targeted therapies

Anaplastic lymphoma kinase (ALK) represents the most clinically actionable genomic alteration currently identified in neuroblastoma (24, 25). ALK mutations and amplification drive tumorigenesis through constitutive activation of the PI3K/AKT/mTOR, RAS/MAPK, and JAK/STAT signaling pathways (26, 27), making the development of ALK inhibitors a significant milestone in precision neuroblastoma therapeutics.

Early-generation ALK inhibitors such as crizotinib showed limited activity in ALK-mutated neuroblastoma, primarily due to incomplete target inhibition and emergence of resistance-associated kinase mutations (26). Next-generation inhibitors, including lorlatinib, have demonstrated more promising efficacy in relapsed and refractory ALK-driven neuroblastoma (28). In a Phase 1 trial, lorlatinib showed significant antitumor activity as monotherapy and in combination with chemotherapy in heavily pretreated pediatric populations (28); further evaluation in Phase 2/3 trials (COG ANBL2232) is ongoing, demonstrating the clinical value of molecularly guided therapy selection. Reported activity in this setting included objective tumor responses, among them complete responses, in a subset of patients with ALK-driven relapsed or refractory disease, although responses were not universal and long-term durability data remain limited (28).

Beyond ALK inhibition, increasing attention is being paid to targeting MYCN-associated oncogenic dependencies (20). Direct MYCN inhibition is technically challenging, but several indirect approaches — including targeting MYCN transcriptional regulation, metabolic reprogramming, and synthetic-lethality vulnerabilities — are being actively pursued (13, 14). BET bromodomain inhibition has emerged as a promising epigenetic strategy to disrupt MYCN-driven transcriptional circuitry and arrest tumor growth (22).

#### 5.1.2 Epigenetic therapies

Epigenetic dysregulation is increasingly recognized as a central driver of neuroblastoma progression, lineage plasticity, and therapeutic resistance (15, 45). Inhibitors of histone deacetylases (HDACs), DNA methyltransferases, and bromodomain and extraterminal (BET) proteins are being evaluated in preclinical and early-phase clinical trials (22). BET inhibitors suppress MYCN-related transcriptional programs and disrupt super-enhancer-mediated oncogenic circuitry (13, 45). Similarly, EZH2-directed therapies may reverse stemness-related epigenetic states and enhance tumor differentiation.

Super-enhancer biology represents another key therapeutic vulnerability in neuroblastoma (43, 45). Lineage-specific enhancer landscapes sustain distinct adrenergic and mesenchymal transcriptional states that can dynamically interconvert under therapeutic pressure (15, 36, 43), suggesting that epigenetic reprogramming directly contributes to relapse and therapy resistance.

#### 5.1.3 Differentiation therapy

Differentiation therapy represents one of the earliest successful examples of biologically driven treatment in neuroblastoma (4). Retinoid-based agents, particularly isotretinoin, induce terminal differentiation of residual neuroblastoma cells and are routinely incorporated into maintenance therapy for high-risk disease (9, 46). Isotretinoin entered high-risk maintenance after randomized data showed an event-free survival benefit for 13-cis-retinoic acid following myeloablative consolidation, and it was subsequently combined with anti-GD2 immunotherapy in the Children’s Oncology Group ANBL0032 regimen (46). Differentiation-inducing approaches aim to overcome developmental arrest and re-establish normal sympathoadrenal maturation pathways (5). Recent molecular studies suggest that modulation of transcriptional regulatory circuitry — including PHOX2B, HAND2, and GATA3 — may further improve differentiation-associated therapeutic responses (13, 15).

#### 5.1.4 Synthetic lethality strategies

Synthetic lethality-based therapeutic strategies targeting tumor-specific vulnerabilities are increasingly explored (14). MYCN-amplified neuroblastomas exhibit profound metabolic dependency, replication stress, and DNA damage response dysregulation (14, 20), rendering tumors potentially sensitive to inhibitors of PARP, ATR, CHK1, aurora kinases, and cell cycle regulatory pathways. Metabolic targeting has also been proposed as an attractive therapeutic strategy, given the increased glycolysis, glutamine dependence, and mitochondrial adaptation characteristic of MYCN-driven tumors (14). Such approaches are emblematic of the broader shift from traditional cytotoxic therapy toward biology-driven precision oncology.

### 5.2 Immunotherapeutic advances

Immunotherapy has transformed the therapeutic landscape of high-risk neuroblastoma and is now a pillar of modern treatment strategies (31). Advances in understanding tumor-associated immunosuppression, antigen expression, and microenvironmental regulation have enabled the development of increasingly sophisticated immunotherapeutic platforms.

#### 5.2.1 Anti-GD2 monoclonal antibodies

Disialoganglioside GD2 is highly expressed on neuroblastoma cells and is limited in normal tissues, making it an excellent immunotherapy target (31). Anti-GD2 monoclonal antibodies such as dinutuximab and naxitamab, now standard of care in high-risk disease, have produced meaningful improvements in survival (2). The pivotal COG trial (Yu et al., 2010) demonstrated that adding dinutuximab with GM-CSF, interleukin-2, and isotretinoin to standard therapy significantly improved event-free and overall survival in high-risk neuroblastoma (46). In that randomized trial, immunotherapy raised 2-year event-free survival from approximately 46% to 66% (p = 0.01) and 2-year overall survival from approximately 75% to 86% relative to standard maintenance (46). These antibodies mediate antitumor effects via antibody-dependent cellular cytotoxicity, complement activation, and immune-mediated tumor destruction (31).

Therapeutic resistance, however, remains a major limitation. Incomplete responses and relapse are substantially attributable to TME-mediated immune suppression, macrophage polarization, T-cell exhaustion, and NK-cell dysfunction (31, 44). Current investigations focus on combination immunotherapy approaches that incorporate checkpoint blockade, cytokine modulation, and cellular therapies.

#### 5.2.2 CAR-T cell therapy

Chimeric antigen receptor T-cell (CAR-T) therapy is among the most promising emerging immunotherapies for neuroblastoma (2). Early clinical trials of GD2-directed CAR-T cells — including NCT02107963 and NCT01822652 — have demonstrated encouraging activity in relapsed and refractory disease, though substantial challenges remain. These include impaired T-cell infiltration into solid tumors, antigen heterogeneity, an immunosuppressive TME, and T-cell exhaustion (31). Next-generation CAR-T strategies focus on improving persistence, overcoming immunosuppression, and enhancing intratumoral infiltration.

#### 5.2.3 NK cell therapy

Natural killer (NK) cells are key mediators of anti-GD2-induced cytotoxicity and represent another promising therapeutic platform (31). Impaired NK-cell function is frequently observed in aggressive neuroblastoma and contributes to immune escape (44). Adoptive NK-cell transfer, cytokine-activated NK-cell therapy, and engineered NK-cell platforms are increasingly explored in combination with anti-GD2 antibodies and checkpoint inhibitors (2).

#### 5.2.4 Bispecific antibodies and vaccine-based approaches

Bispecific antibodies targeting tumor-associated antigens and immune effector cells simultaneously have shown promising preclinical activity (2). These agents may enhance immune synapse formation and boost cytotoxic responses in immunosuppressive TMEs. Therapeutic vaccine approaches are also under investigation to generate robust tumor-specific immune responses (31). Dendritic cell-, peptide-, and neoantigen-targeted platforms aim to stimulate adaptive antitumor immunity and reduce the risk of recurrence.

### 5.3 Multi-omics guided personalized oncology

The emergence of multi-omics technologies has transformed neuroblastoma research and therapeutic design (10). Integration of genomic, transcriptomic, epigenomic, proteomic, metabolomic, radiomic, and liquid biopsy data allows increasingly precise characterization of tumor biology and clonal evolution.

#### 5.3.1 Precision trial design

Precision oncology has enabled the development of biomarker-driven clinical trial platforms incorporating real-time molecular profiling for therapeutic selection (2). Modern trial designs are increasingly stratifying patients based on genomic alterations, including ALK mutations, MYCN amplification, telomere maintenance status, and immune-related signatures (24, 28). Given significant intratumoral heterogeneity and evolving molecular landscapes, adaptive basket and umbrella trial models are particularly well-suited to neuroblastoma (2), enabling evaluation of targeted therapies in biologically defined patient subsets rather than relying on conventional histological classification.

#### 5.3.2 Adaptive therapy models

Therapeutic resistance and tumor dynamics are major challenges in high-risk neuroblastoma (35). Longitudinal genomic profiling has revealed substantial mutational heterogeneity between primary and relapsed tumors, underscoring the need for adaptive therapeutic strategies (35). Adaptive therapy models aim to modify treatment intensity and combinations in response to evolving tumor biology (2). Serial molecular surveillance, combining radiogenomics and liquid biopsy, may enable earlier detection of resistant clones and mechanisms of therapeutic escape (37, 38).

#### 5.3.3 Dynamic biomarker monitoring

Liquid biopsy technologies have become valuable tools for real-time biomarker monitoring (11, 40). Circulating tumor DNA, CTCs, exosomal biomarkers, and cell-free RNA provide minimally invasive opportunities for longitudinal assessment of tumor evolution and therapeutic response (38–40). Detection of ALK mutations, MYCN amplification, and emerging resistance-associated alterations in circulating nucleic acids may enable earlier intervention and personalized treatment modification (39, 40), while more comprehensively representing intratumoral heterogeneity than single-site tissue biopsy.

#### 5.3.4 Real-time treatment modification

Artificial intelligence, machine learning, and multi-omics analytics are increasingly enabling real-time treatment optimization (41). AI-powered prediction systems can integrate imaging, molecular, and clinical data to stratify high-risk patients, predict therapeutic response, and guide the adaptation of personalized treatment (41, 42). Radiogenomic approaches linking imaging phenotypes to molecular features further support non-invasive precision oncology (42) and may, in the future, enable dynamic treatment adjustments based on biological signatures that evolve over the disease course.

Translational integration of genomics, immunotherapy, epigenetics, liquid biopsy, and artificial intelligence is collectively redefining neuroblastoma therapeutics and driving the transition toward truly individualized pediatric precision oncology.

Current and emerging precision therapeutic strategies in neuroblastoma are summarized in **Table 4**.

**Table 4:** Major precision therapeutic strategies in neuroblastoma.

Therapeutic strategy	Target/mechanism	Representative agents	Current clinical status	References
ALK inhibition	ALK signaling blockade	Lorlatinib, crizotinib	Clinical trials/approved use in refractory disease	(28)
Anti-GD2 immunotherapy	GD2 surface antigen targeting	Dinutuximab, naxitamab	Standard of care in high-risk disease	(31, 46)
Differentiation therapy	Promotion of tumor maturation	Isotretinoin	Maintenance therapy (standard of care)	(4, 9, 46)
Epigenetic therapy	Chromatin/transcriptional modulation	BET inhibitors, HDAC inhibitors	Early-phase clinical trials	(13, 22, 45)
CAR-T cell therapy	Engineered T-cell targeting	GD2 CAR-T platforms	Investigational	(2, 31)
NK-cell therapy	Enhanced innate immunity	Cytokine-activated NK cells	Investigational	(2, 31)
Bispecific antibodies	Simultaneous tumor and immune targeting	GD2-directed bispecific constructs	Early development	(2)
Synthetic lethality strategies	Exploitation of DNA repair vulnerabilities	PARP inhibitors, ATR inhibitors	Preclinical/early clinical evaluation	(14, 20)
Liquid biopsy-guided therapy	Dynamic molecular monitoring	ctDNA-guided adaptive therapy	Emerging clinical application	(38, 40)

ALK, anaplastic lymphoma kinase; ATR, ataxia telangiectasia and Rad3-related protein; BET, bromodomain and extraterminal domain; CAR-T, chimeric antigen receptor T-cell; ctDNA, circulating tumor DNA; GD2, disialoganglioside GD2; HDAC, histone deacetylase; NK cell, natural killer cell; PARP, poly(ADP-ribose) polymerase. Therapeutic approaches summarized include currently approved therapies and emerging translational strategies under active preclinical or clinical investigation in precision neuroblastoma oncology (2, 4, 9, 13, 22, 28, 31, 46).

## 6 Current limitations and challenges

Despite major advances in neuroblastoma genomics, immunotherapy, and precision oncology, several biological, technological, logistical, and ethical challenges continue to impede successful clinical translation (2, 5). The extraordinary biological heterogeneity of neuroblastoma remains a central obstacle to durable therapeutic success and precise prognostic prediction (7, 15).

### 6.1 Tumor heterogeneity

Neuroblastoma exhibits substantial intertumoral and intratumoral heterogeneity at the genomic, epigenomic, transcriptomic, metabolic, and microenvironmental levels (7, 15). Within individual tumors, distinct adrenergic and mesenchymal cellular states may coexist and dynamically interconvert under therapeutic pressure (36, 43). This lineage plasticity is a major driver of treatment resistance, metastatic spread, and disease relapse (35). Single-cell sequencing studies have demonstrated the presence of resistant subclonal populations that survive chemotherapy and seed recurrence (10), while longitudinal genomic analyses reveal considerable mutational divergence between primary and relapsed tumors (35). Single-site tissue biopsy frequently fails to capture the full biological complexity of the disease.

### 6.2 Small pediatric datasets

Pediatric cancers are rare compared with adult malignancies, limiting the availability of large-scale datasets for biomarker validation and machine-learning model development (1, 2). Neuroblastoma’s biological heterogeneity further reduces effective sample sizes available for subgroup-specific analyses (7). Small cohort sizes frequently limit statistical power and reproducibility in multi-omics investigations, particularly for rare genomic alterations and relapsed disease populations (2). Low incidence also complicates prospective clinical trial design and the validation of precision therapeutic strategies (28).

### 6.3 Limited validation cohorts

Despite the identification of numerous candidate biomarkers, rigorous multicenter validation has been achieved for only a small subset (2). Many biomarkers derived from transcriptomics, epigenetics, radiomics, and AI approaches remain confined to retrospective studies and have not been externally reproduced (41, 42). Inconsistencies across studies frequently arise from differences in sequencing platforms, imaging protocols, computational approaches, and patient selection criteria (2). Harmonized validation frameworks and international collaborative datasets are therefore essential for wider clinical translation.

### 6.4 Cost and accessibility

Comprehensive molecular profiling, single-cell sequencing, spatial transcriptomics, and advanced radiogenomic analyses remain expensive and technologically demanding (10, 41). Access to these technologies varies widely by region and healthcare system, especially in low- and middle-income countries where neuroblastoma-related mortality remains disproportionately high (1). Similarly, many novel targeted and immunotherapeutic agents — including anti-GD2 monoclonal antibodies, CAR-T cell therapies, and next-generation ALK inhibitors — carry a significant financial burden (28, 31), raising critical concerns about equitable access to precision pediatric oncology.

### 6.5 Standardization and data integration challenges

The lack of methodological standardization is a major barrier to translational neuroblastoma research (2). Variations in tissue processing, sequencing methods, liquid biopsy assays, radiomic feature extraction, and bioinformatic analysis pipelines critically affect the reproducibility and comparability of studies (11, 40). Radiomics and AI-based models are especially vulnerable to standardization issues, as imaging acquisition parameters, segmentation methods, and computational architectures can significantly affect prediction performance (41, 42). Establishing internationally harmonized protocols and quality-control frameworks is therefore essential.

The integration of large-scale multidimensional datasets spanning genomic, transcriptomic, epigenomic, proteomic, metabolomic, radiomic, and clinical domains introduces additional computational complexity (2). High-dimensional data interpretation requires advanced computational infrastructure, sophisticated machine learning approaches, and interdisciplinary collaboration across oncologists, bioinformaticians, radiologists, pathologists, and systems biologists (41). Biological correlations identified in multi-omics datasets do not always translate directly into clinically actionable therapeutic strategies and therefore require careful validation before implementation.

### 6.6 Ethical considerations in genomic testing

The increasing application of genomic and germline testing in pediatric oncology presents important ethical considerations (3, 34). Recognition of hereditary predisposition syndromes involving ALK or PHOX2B mutations may have profound implications for family counseling, surveillance strategies, reproductive decision-making, and psychosocial well-being (3, 12, 24). Complex issues surrounding informed consent, incidental findings, genomic privacy, long-term data storage, and pediatric assent remain active areas of ethical deliberation (3). Ensuring that informed consent procedures align with established guidelines — including those from the European Society of Human Genetics (ESHG) and the International Society of Nurses in Genetics (ISONG) — is essential, as is compliance with applicable data protection frameworks (e.g., GDPR for European datasets). Furthermore, equitable access to genomic testing and advanced therapies across diverse socioeconomic and geographic settings remains an ongoing priority in global pediatric oncology frameworks.

These limitations collectively underscore the necessity of robust multicenter collaboration, standardized translational pipelines, equitable access to healthcare, and biologically informed computational frameworks to fully harness the potential of precision neuroblastoma oncology.

### 6.7 Bridging discovery and clinical implementation

Despite rapid advances in molecular characterization and computational oncology, several translational gaps continue to limit the integration of precision neuroblastoma strategies into routine clinical care. Many emerging biomarkers identified through transcriptomic, epigenetic, radiomic, and liquid biopsy studies still lack prospective multicenter validation and reproducibility across independent cohorts.

Clinical implementation is also constrained by limited pediatric datasets, the financial costs of advanced multi-omics technologies, and inequitable access to specialized precision oncology infrastructure across healthcare systems. Ethical concerns regarding genomic privacy, incidental germline findings, and long-term pediatric data storage further complicate implementation.

Future translational progress will depend on international collaborative frameworks, standardized molecular testing protocols, transparent AI validation systems, and adaptive clinical trial designs capable of integrating longitudinal multi-omics surveillance into.

## 7 Future directions

The future management of neuroblastoma is expected to undergo fundamental reshaping through rapid advances in systems biology, computational oncology, immunogenomics, and artificial intelligence (2, 41). Emerging technologies are designed not only to better characterize tumor biology but also to enable dynamic, real-time therapeutic adaptation.

### 7.1 Spatial transcriptomics

Spatial transcriptomics preserves tissue architecture while mapping gene expression within its native microenvironmental context, complementing bulk and single-cell RNA sequencing (10). In principle, it could show where adrenergic and mesenchymal cell states sit relative to stroma and immune infiltrate, and how metastatic niches are organized (15, 44). In practice, however, its contribution to neuroblastoma remains unproven: the available work is largely descriptive, based on small numbers of tumors, and has not yet altered diagnosis, risk assignment, or treatment. The platforms are costly, technically demanding, and sensitive to tissue quality and analytical choices, and no prospective neuroblastoma study has shown that spatial data add decision-relevant information beyond single-cell sequencing. Its value will depend on isolating the specific questions — for example, the spatial immune contexture relevant to GD2-directed therapy — that cheaper, established methods cannot answer.

### 7.2 Proteogenomics

Proteogenomics combines genomic, transcriptomic, and proteomic data on the rationale that protein abundance and post-translational modification may track treatment response more directly than DNA-level alterations (2). This rationale is attractive but, for neuroblastoma specifically, currently aspirational: large-scale tumor proteomes are scarce, pediatric tissue is limited, and no proteogenomic signature has been validated as diagnostic, prognostic, or predictive in this disease. We therefore regard proteogenomics as a hypothesis-generating tool whose clinical relevance in neuroblastoma remains to be demonstrated, most plausibly by clarifying signaling-level adaptive resistance that genomic profiling alone cannot capture (13, 20).

### 7.3 Digital twins in oncology

The concept of the “digital twin” represents an emerging frontier in precision oncology (41). Digital twins are patient-specific computational tumor models that integrate clinical, imaging, genomic, and transcriptomic data, as well as therapeutic response data. Such models have been explored as proof of concept in other cancer settings (e.g., Björnsson et al., 2020) and may eventually enable simulation of disease progression, prediction of treatment response, optimization of treatment sequencing, and individualized toxicity assessment (41, 47). Although digital oncology platforms remain largely experimental, they hold transformative potential for pediatric cancer care.

### 7.4 AI-integrated clinical decision systems

Artificial intelligence is expected to become increasingly integrated into the management of clinical neuroblastoma (41). AI-based decision support systems that integrate imaging, pathology, molecular profiling, and longitudinal clinical data may substantially improve diagnostic accuracy, prognostic precision, and therapeutic optimization. Machine learning algorithms may enable automated risk stratification, relapse prediction, treatment response monitoring, and adaptive therapy selection (41, 42). Importantly, future AI systems are envisioned as clinically assistive tools designed to complement, rather than replace, physician-guided decision-making.

### 7.5 Multi-modal biomarker fusion

Future precision oncology in neuroblastoma will increasingly rely on integrated multi-modal biomarker fusion, combining genomics, epigenomics, transcriptomics, radiomics, liquid biopsy data, immune profiling, and clinical variables (2, 41). Such integrated frameworks may improve prediction of therapeutic response, identification of resistant subclones, and individualized risk stratification, while also enabling dynamic monitoring of disease during treatment (40).

### 7.6 Real-time liquid biopsy-guided therapy

Liquid biopsy technologies are poised to become key tools for real-time therapeutic monitoring (11, 40). Serial assessment of circulating tumor DNA, exosomal biomarkers, and cell-free RNA may allow early identification of therapeutic resistance, clonal evolution, and minimal residual disease (38, 39). Integration of liquid biopsy data into adaptive therapy models may enable treatment change prior to frank clinical relapse (40), ultimately reducing the need for repeated invasive tissue biopsies and improving longitudinal disease surveillance.

### 7.7 Precision immuno-oncology

**Figure 3:**
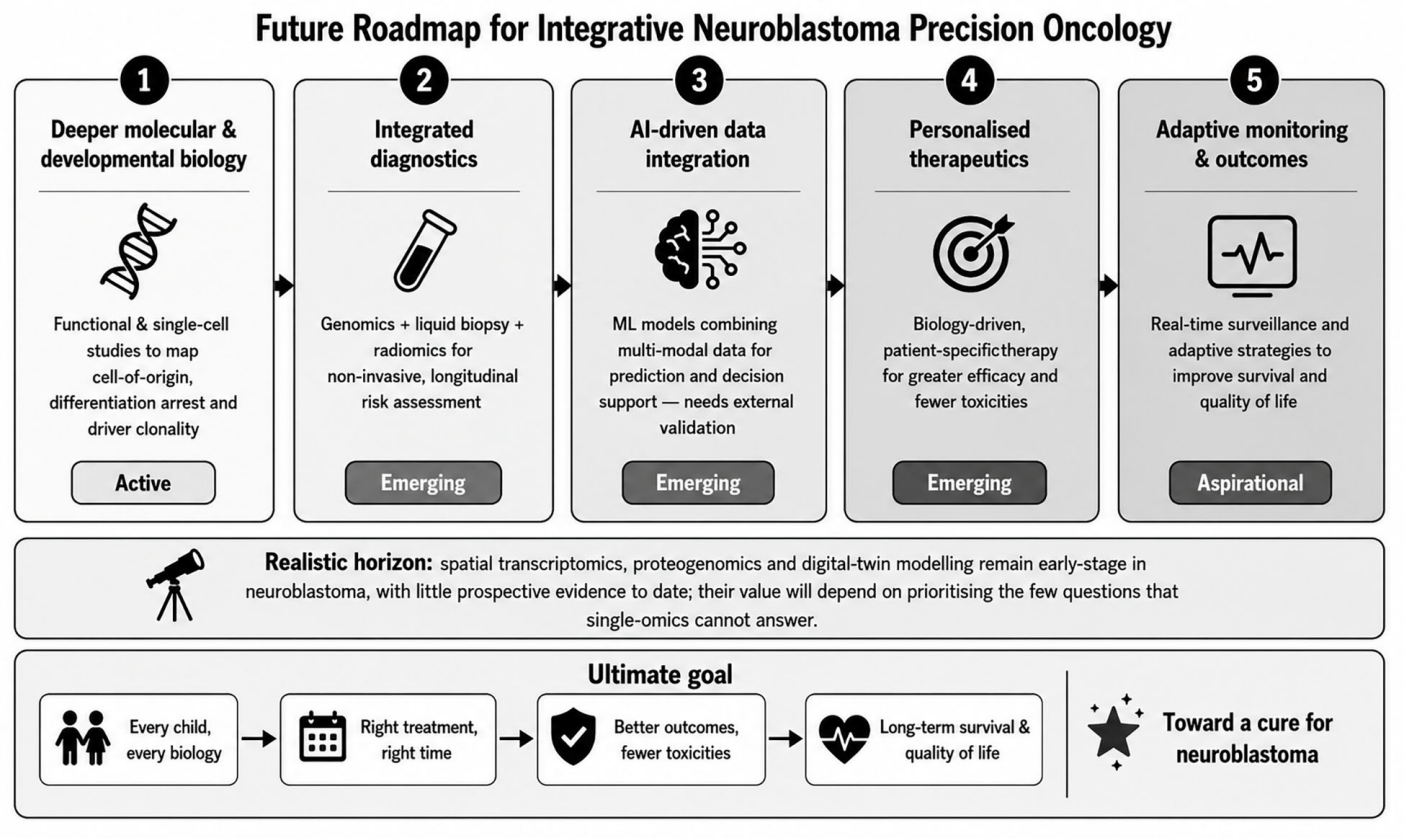
Conceptual roadmap of future directions in integrative neuroblastoma precision oncology. The image illustrates the convergence of spatial transcriptomics, proteogenomics, digital twin modeling, AI-integrated clinical decision systems, real-time liquid biopsy monitoring, and precision immuno-oncology as the next horizon in individualized pediatric cancer care.

Future immunotherapeutic strategies will likely involve more personalized approaches based on tumor genomics, immune microenvironmental profiling, and antigenic heterogeneity (31). Advances in next-generation CAR-T engineering, bispecific antibodies, NK cell platforms, neoantigen vaccines, and immune checkpoint modulation may significantly improve therapeutic efficacy in refractory neuroblastoma (31). Combining immunogenomics with spatial transcriptomics and multi-omics profiling could further help identify patients most likely to benefit from specific immunotherapeutic interventions. These anticipated future directions in integrative neuroblastoma precision oncology are summarized in **Figure 3**.

## 8 Conclusion

Neuroblastoma is one of the most biologically heterogeneous pediatric malignancies, and its management has been profoundly transformed by advances in genomics, epigenetics, immunology, and computational oncology (2, 5, 7). The integration of multi-omics profiling, liquid biopsy, radiomics, and artificial intelligence is fundamentally reshaping diagnostic precision, prognostic stratification, and therapeutic personalization (10, 11, 41). Critically, it is the convergence and cross-platform integration of these approaches — rather than their individual application — that will drive the next wave of advances in precision pediatric oncology.

The promise of molecularly guided therapy is increasingly realized through targeted therapies such as lorlatinib and epigenetic modulators, as well as immunotherapeutic strategies, including anti-GD2 antibodies, CAR-T cells, and NK-cell therapies (28, 31, 46). Nevertheless, tumor heterogeneity, clonal evolution, scarcity of pediatric datasets, and inequities in access to advanced diagnostics and therapeutics continue to hinder clinical translation (2, 35).

Future integration of spatial transcriptomics, proteogenomics, radiogenomics, and real-time biomarker-guided adaptive therapy is expected to further advance precision neuroblastoma oncology, ultimately enabling more effective, less toxic, and genuinely personalized care for children with this challenging disease (10, 40, 41).
